# Biodiversity of Pathogenic and Toxigenic Seed-Borne Mycoflora of Wheat in Egypt and Their Correlations with Weather Variables

**DOI:** 10.3390/biology10101025

**Published:** 2021-10-11

**Authors:** Yasser M. Shabana, Younes M. Rashad, Khalid M. Ghoneem, Nehal S. Arafat, Dalia G. Aseel, Aiming Qi, Benjamin Richard, Bruce D. L. Fitt

**Affiliations:** 1 Plant Pathology Department, Faculty of Agriculture, Mansoura University, Mansoura 35516, Egypt; ymsh@mans.edu.eg (Y.M.S.); nohamaged@mans.edu.eg (N.S.A.); 2 Plant Protection and Biomolecular Diagnosis Department, Arid Lands Cultivation Research Institute (ALCRI), City of Scientific Research and Technological Applications (SRTA-City), New Borg El-Arab 21934, Egypt; daseel@srtacity.sci.eg; 3 Department of Seed Pathology Research, Plant Pathology Research Institute, Agricultural Research Center, Giza 12112, Egypt; khalid_ghoneem@yahoo.com; 4 School of Life and Medical Sciences, University of Hertfordshire, Hatfield AL10 9AB, UK; a.qi@herts.ac.uk (A.Q.); b.richard2@herts.ac.uk (B.R.); b.fitt@herts.ac.uk (B.D.L.F.)

**Keywords:** biodiversity, climate change, phylogeny, seed-borne fungi, *Ustilago tritici*, *Tilletia tritici*, wheat

## Abstract

**Simple Summary:**

Pathogenic fungi cause yield and quality losses and threaten food security. In this study, 198 samples of wheat grains, representing 20 Egyptian wheat cultivars, were collected from 25 wheat-growing governorates across Egypt, and screened for their seed-borne fungi. Twenty genera and 44 species of seed-borne fungi were identified, and their biodiversity indicators and evolutionary relationships were studied based upon similarities in their genetic characteristics. The most frequent fungi were *Alternaria* *alternata* and *Cladosporium* spp., while *Tilletia tritici* and *Ustilago tritici* were the most common smut fungi. The highest fungal diversity was recorded for Sinai governorate, while the greatest species richness was recorded in Qena and Sohag governorates. Correlations of the detected fungi with weather variables (temperature, relative humidity, precipitation, wind speed, or solar radiation) were investigated. Our results indicated that the relative humidity was the most influential weather variable, followed by temperature, solar radiation, wind speed, and precipitation, respectively. Despite this study being conducted on the wheat-growing areas in Egypt, our findings are useful for other wheat-growing countries that share the same climatic conditions. The correlation between a given fungus and the climatic variables can be useful in other ecosystems.

**Abstract:**

Surveillance investigations for pathogenic and toxigenic fungi are important to refine our understanding of their epidemiology and help in predicting their outbreaks. During 2019, 198 samples of wheat grains were collected from 25 wheat-growing governorates in Egypt to detect and identify seed-borne mycoflora in vitro. Forty-four fungal species belonging to 20 genera were identified. Molecular data for these fungi were analyzed to construct a phylogenetic tree. Occurrence and biodiversity indicators were calculated. Two prevalent pathogens (average incidence > 40%) were *Alternaria alternata* and *Cladosporium* spp. *Ustilago tritici* was present in only seven of the 25 governorates, and less abundant than *Tilletia tritici*, the causal agent of stinking smut. Sinai governorate recorded the greatest species diversity, while the greatest species richness was in Qena and Sohag governorates. Canonical correspondence analysis of data for 20 fungal genera with temperature, relative humidity, precipitation, wind speed or solar radiation revealed that relative humidity was the most influential weather variable. It showed that occurrence and distribution of the 20 genera corresponded well with three out of four Egyptian climatic regions: Mediterranean, semi-arid, and arid. Knowing pathogen occurrence and distribution in Egypt is the first step to developing future disease management strategies to limit yield losses and improve food security. Despite this study being conducted on the wheat-growing areas in Egypt, our findings are useful for other wheat-growing countries that share the same climatic conditions. The correlation between a given fungus and the climatic variables can be useful in other ecosystems.

## 1. Introduction

Wheat (*Triticum aestivum* L.) is one of the major cereal crops grown in the world. In Egypt, it ranks first in terms of food security and cultivated area, providing a staple food for more than 100 million people. In 2019, the wheat-cropping area in Egypt was 1,410,912 hectares, accounting for about 50% of the total winter cultivated area and producing a yield of 9 million tons. In addition, Egypt imported c. 13 million tons of wheat in 2019/2020 and was the largest importer of wheat in the world [1]. Wheat grains are exposed to a wide array of pathogenic and toxigenic fungi, which adversely affect seed germination, plant health and yield, and grain nutritional and marketing values [2]. The yield loss in wheat due to fungal infection may be up to 50% if pathogen incidence is great [3]. Moreover, a set of seed-borne fungi, mainly members of the genera *Aspergillus*, *Penicillium*, *Fusarium*, and *Alternaria*, is toxigenic, which results in many deleterious effects on human and livestock health, in addition to considerable economic losses in the agriculture and food industry sectors [4]. Mycoflora associated with wheat grain are found on/in the seed coat, embryo, or endosperm [5]. Many seed-borne fungi have been reported on wheat grain in various countries. The most common fungi reported on wheat include *Fusarium* spp., *Bipolaris* spp., *Alternaria* spp., *Curvularia* spp., *Aspergillus* spp., and *Penicillium* spp. [6]. In addition, the smut fungi, *Ustilago tritici* and *Tilletia tritici*, have also been reported [7,8]. In Egypt, several studies on the seed-borne fungi of wheat have been completed. El-Kady et al. [9] reported 25 genera and 59 species of seed-borne fungi on wheat grains collected from various sites in Egypt, with the highest dominance of the genus *Aspergillus* (18 species + 2 varieties), followed by *Penicillium* (12 species + 1 variety). The genus *Fusarium* came third in this regard (5 species + 1 variety), followed by *Rhizopus* spp., *Mucor* spp., *Alternaria* spp., and *Curvularia* spp. with two species for each, while the other fungi (one species for each) were detected in very minor incidence. Mazen et al. [10] identified 28 genera and 72 species of seed-borne fungi from Egyptian wheat grains. The most common species were *A. niger, A. flavus, A. terreus, A. nidulans, A. alternata, Cladosporium herbarum*, and *F. oxysporum*. Another study was conducted to detect wheat seed-borne mycoflora from 14 Egyptian wheat cultivars by Baka [11]. The most frequent fungi were *A. flavus*, *A. niger*, *A. alternata*, and *F. verticillioides*.

Environmental conditions have a great importance in plant pathology, forming the classic disease triangle along with the pathogen and the host [12]. Extreme fluctuation in the climatic conditions, even for a short period, may severely affect growth, survival, resistance, and health of plants [13]. In addition, climate change may suppress/enhance the growth, toxigenicity, virulence, host specificity, reproduction, dissemination, or geographic distribution of the pathogen, influencing disease severity and spread [14,15]. Weather variables such as temperature, relative humidity, rainfall, and wind speed can positively or negatively affect plant-pathogen interactions [16]. A recent study investigated the impact of climate change on the future distribution of *F. oxysporum* in north Africa, the Middle East, and Europe [17]. The results indicated that, while the pathogen distribution will increase in some regions, it is predicted to reach severe epidemic levels in other regions by 2050 and 2100. Potential disease outbreaks due to climate change make it imperative to understand the correlations between the pathogen and the weather variables to predict future pathogen dissemination in order to take appropriate decisions [18]. Understanding the influence of weather variables on pathogen behavior can lead to changes in disease control measures, and assessment of feasibility, suitability, and timing of crop cultivation in particular regions. This work was planned to detect/identify the seed-borne mycoflora of wheat across all wheat-growing governorates in Egypt, to analyze their biodiversity indicators and geographical distribution, to study their phylogenetic relationships and clustering structure, and to investigate their correlations with different weather variables. This work is a primary study in a long-term joint project between scientists in UK and in Egypt, which aims to predict the impacts of climate change on future distribution and severity of pathogenic seed-borne fungi on wheat in Egypt up to 2100. Based on predictions, adaptation strategies and policies will be recommended for government research and development programs to mitigate negative impacts of both climate change and crop diseases.

## 2. Materials and Methods

### 2.1. Study Area

Twenty-five wheat-growing governorates in Egypt were surveyed for the 2018/2019 wheat cropping season during 2019 (April and May). The study area was located between latitudes 22°00′ N and 31°50′ N, and longitudes 25°00′ E and 35°00′ E. The surveyed area represented diverse climatic conditions.

### 2.2. Meteorological Conditions

In general, Egypt has an arid to semi-arid climate; however, there are four distinctive climatic regions in Egypt. They are Mediterranean on the northern coast, semi-arid in the middle of the country, arid in the southern areas, and Red Sea climate on the eastern coast. Egypt is mostly a subtropical area, but the southern part is tropical. Mainly, winds blow from the north coast throughout the year, moderating temperature in the northern areas, while in the southern areas, a hot, dry, dusty wind, known as Khamaseen, blows from the Sahara Desert in March to May, increasing temperature to 50 °C, while relative humidity can decrease to <5%. The annual average rainfall across the country ranges from 20 to 200 mm, decreasing gradually from the north to the south. However, in many southern arid areas, it may rain once in many years. During the wheat-growing season (from November 2018 to May 2019), the mean air temperature ranged from 16 to 21 °C, the relative humidity ranged from 33.2 to 71.3%, and the rainfall ranged from 0 to 10.0 mm [19].

### 2.3. Sample Collection

**Figure 1:**
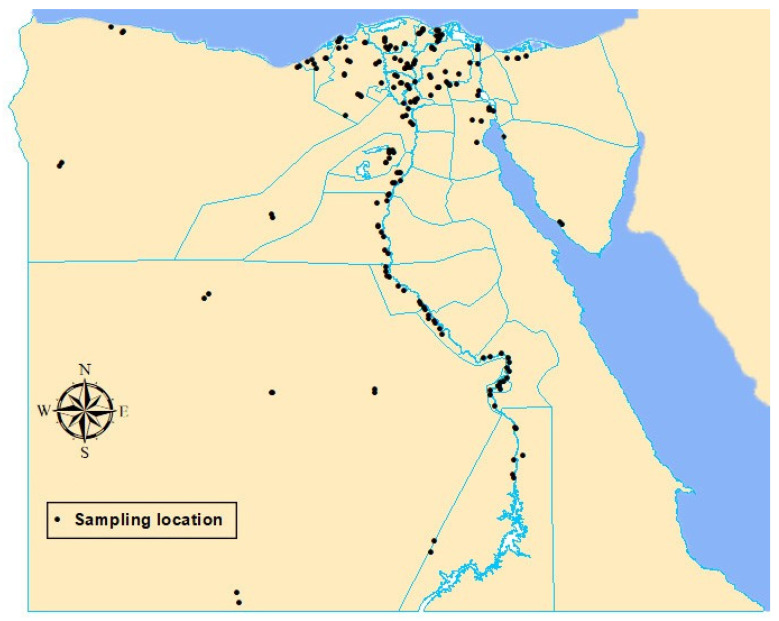
Wheat grain sampling locations (black dots) in the 25 wheat-growing governorates of Egypt.

For each governorate, four districts were selected to represent the wheat-growing areas in the governorate with at least 15 km between them. For each district, two villages in opposite directions with at least 5 km between them were selected, and one wheat-cropping field from each village was sampled. In Egypt, the wheat-grown areas represent approximately 4% of the total territory area of the country and are mainly concentrated on the Nile delta and Nile valley. Most of the surveyed governorates are adjacent to each other and the boarders between them are not separated. Furthermore, most of these adjacent governorates share the same weather conditions. Therefore, a 15-km distance between two districts and a 5-km distance between two villages are suitable. Each sample was composed of sub-samples that were taken in a random zigzag pattern from the wheat-cropping field and c. 1 kg of grain was collected and kept in a paper bag, labeled, and stored in a cool box (at ~10 °C) until arrival at the laboratory, then stored in a refrigerator at 4 °C until use within 1 to 4 days. For each sampling site, the field information was recorded, and the location was georeferenced using the global positioning system (GPS). These spatial coordinates were used to show the wheat grain sampling locations on a map (Figure 1).

### 2.4. Detection of Seed-Borne Fungi

For detection of seed-borne fungi, three seed-health techniques recommended by the International Seed Testing Association were used, namely the deep-freezing blotter method (DFB), washing test, and embryo count test [20].

#### 2.4.1. Deep-Freezing Blotter Method (DFB)

For each sample, 400 grains were plated in 9-cm diameter sterile Petri dishes containing three layers of sterile blotting paper moistened with sterilized tap water at 25 grains per Petri dish. The plates were then incubated at 21 ± 2 °C for 24 h and then transferred to a −20 °C freezer for 24 h. After this, the plates were incubated at 20 ± 2 °C for 5 days under cool white fluorescent light with alternating cycles of 12 h light/12 h darkness. Frequency and incidence percentages of each fungus species identified were calculated according to the following equations:

(1) $$\mathrm{Frequency}\;\left(\%\right)=\frac{\mathrm{Number}\;\mathrm{of}\;\mathrm{the}\;\mathrm{fungus-infected}\;\mathrm{samples}}{\mathrm{Total}\;\mathrm{number}\;\mathrm{of}\;\mathrm{samples}\;\mathrm{tested}}\times 100$$

(2) $$\mathrm{Incidence}\;\left(\%\right)=\frac{\mathrm{Number}\;\mathrm{of}\;\mathrm{the}\;\mathrm{fungus-infected}\;\mathrm{grains}}{\mathrm{Total}\;\mathrm{number}\;\mathrm{of}\;\mathrm{grains}\;\mathrm{tested}}\times 100$$

For the smut fungi, *T. tritici* and *U. tritici*, the density of teliospores (for *T. tritici*) or infected embryos (for *U. tritici*) was used instead of incidence. The smut fungus density was the number of teliospores per 100 g of grains, indicating inoculum concentration in the grain, which was counted from the washing test (see below) for *T. tritici*, and the number of embryos infected per 100 g of grains was counted using the embryo count technique (see below) for *U. tritici*.

#### 2.4.2. Washing Test

This was used for detection of seed-borne smut fungi, except the loose smut fungus (*U. tritici*). For each sample, 100 g of wheat grains were transferred to an Erlenmeyer flask (250 mL) containing 100 mL of sterile water and amended with 1–2 drops of Tween 20. The flask was kept on an orbital shaker at 150 rpm for 5–10 min, after which the flask content was filtered through a piece of cheesecloth and the washing solution was collected. The filtered washing solution was then centrifuged at 3000 rpm for 2–10 min. Supernatant was carefully discarded and the tubes were kept inverted, while the tube edge was wiped with a paper towel to absorb the remaining drops. One milliliter of sterile water was then added to the pellet, and the tube content was mixed using a needle to form a homogenized spore suspension. Two or three single drops of the suspension were mounted on a hemocytometer slide and then examined under a light microscope. The total number of spores per 100 g of wheat grains was determined.

#### 2.4.3. Embryo Count Test

This was used for the detection of seed-borne loose smut fungus, *U. tritici*. For each sample, 100 g of wheat grains was soaked in 1L of freshly prepared sodium hydroxide solution (5%) and incubated at 22 ± 2 °C for 24 h. The soaked grains were then washed using running tap water to extract the embryo through the ruptured pericarps. A set of three sieves (3.5, 0.2, and 0.1 mm mesh diameter) were used to extract the embryos from the grain suspensions using gently running water. The embryos collected were then transferred to a 250 mL beaker containing 75 mL of a mixture of lactic acid, phenol, glycerol, and water (1:1:2:1), and heated until boiling for 2 min. The lactic acid-glycerol mixture was allowed to cool, and then the embryos were examined.

### 2.5. Identification of Seed-Borne Fungi

Morphological identification of the seed-borne fungi was achieved using their cultural properties and their macro- and microscopic characteristics according to Domsch et al. [21] and Summerell et al. [22]. For molecular identification, DNA of the fungi isolated was extracted according to the manual of the DNA extraction kit (Qiagene, Germany). The internal transcribed spacer (ITS) region (600 bp) was amplified according to the method described by White et al. [23] with modifications using the primers ITS1 (5′TCCGTAGGTGAACCTTGCGG3′) and ITS4 (5′TCCTCCGCTTATTGATATGC3′). The PCR products were purified using a gel extraction purification kit (Maxim Biotech INC, Rockville, MD, USA) and sequenced (Macrogene Company, Seoul, Korea). The nucleotide sequences were aligned using the ClustalW algorithm and identified by comparing the available sequences in the GenBank database using the NCBI search tool BLAST. The phylogenetic tree of the fungal species identified was constructed by the maximum likelihood method, using MEGA X software version 10.2.4 [24].

### 2.6. Calculating Biodiversity Indicators

Diversity of the fungal species isolated in the wheat-cropping governorates of Egypt surveyed was studied. Frequency and relative abundance were determined at a national scale using grain sampling and seed testing data aggregated across all 25 wheat-growing governorates. The relative abundance was calculated:

(3) $$\mathrm{Relative}\;\mathrm{abundance}\;\left(\%\right)=\frac{\mathrm{Number}\;\mathrm{of}\;\mathrm{grains}\;\mathrm{infected}\;\mathrm{with}\;\mathrm{the}\;\mathrm{given}\;\mathrm{fungus}\;\mathrm{species}}{\mathrm{Total}\;\mathrm{number}\;\mathrm{of}\;\mathrm{grains}\;\mathrm{infected}\;\mathrm{with}\;\mathrm{all}\;\mathrm{fungus}\;\mathrm{species}\;\mathrm{identified}}\times 100$$

The values of species richness and the Shannon–Wiener diversity index were determined in each of the 25 wheat-growing governorates as follows:

Species richness = total number of fungus species identified in a wheat-growing governorate.

The Shannon–Wiener diversity index was calculated using the following equation:

(4) $$\mathrm{Shannon}\text{–}\mathrm{Wienerdiversity}\;\mathrm{index}\;\left(H\right)\;=-\overset{s}{\underset{i=1}{\sum }}\;P_{i}\times Ln\left(P_{i}\right)\;$$

where *P_i_* = ni/N (ni is the number of grains with the species identified i and N is the total number of grains with all fungus species identified), which is the relative abundance expressed in a fractional form.

Distribution and incidence of the important pathogenic seed-borne fungi of wheat were geographically mapped using the software and the packages “raster”, “sp”, and “ggplot2”.

### 2.7. Pathogenicity Test

Fifteen seed-borne fungi were isolated, namely; *A. alternata*, *B. austrostipae*, *B. cynodontis*, *B. sorokiniana*, *B. tetramera*, *C. anthropophilum*, *C. mebaldsii*, *Exerohilum rostratum*, *F. chlamydosporum*, *F. equiseti*, *F. fujikuroi*, *F. oxysporum*, *F. proliferatum*, *F. verticillioides*, and *Stemphylium globuliferum*, and were tested for their pathogenicity using a soil infestation technique. For inoculum preparation, the fungi tested were cultured on sterilized sorghum–sand medium (1:1) at 10% moisture for 15 d at 26 ± 2 °C. Pots (25 cm-diameter) filled with sterilized soil were individually infested with the fungal inoculum at a rate of 0.3% (*w*/*w*) (i.e., 3 g of inoculum was added with every 1000 g of soil). The soil was then mixed thoroughly, watered with tap water, and kept moistened for one week before planting. In each pot, 10 surface-sterilized wheat grains were sown. For each fungus, 10 pots (replicates) were used. All pots were arranged in a completely randomized design and kept for two months in a greenhouse. The pots were observed daily for grain germination and seedling damping-off. The disease incidence and mortality rate were then determined.

### 2.8. Statistical Analyses

The data were first examined for normality and then subjected to analysis of variance (ANOVA). Comparison of means was performed with Duncan’s multiple range test [25] at *p* ≤ 0.05 based on one-way ANOVA using the statistical analysis software “CoStat 6.4” [26]. Correlations between the incidences of seed-borne fungi and weather variables were indicated on the ordination diagram produced by Canonical Correspondence Analysis (CCA) using the software R and the package “vegan”. The weather variables included daily mean air temperature (°C), relative humidity (%), and wind speed (km/h), as well as the monthly mean precipitation (mm) and solar radiation (kWh/m2) during November 2018 to April 2019 in each governorate sampled.

## 3. Results

### 3.1. Occurrence and Distribution of Wheat Seed-Borne Fungi

Seed-borne fungi of wheat were detected and identified in samples across all wheat-growing governorates of Egypt for the 2018/2019 cropping season. Two hundred samples of wheat grains were collected from 25 wheat-growing governorates (Figure 1), but two samples were lost during transportation. Therefore, results were based on one hundred and ninety-eight samples. The collected wheat samples represented twenty types of the Egyptian wheat cultivars which were grown across the sampled wheat-growing fields, namely, Giza 168, Giza 169, Giza 171, Misr 1, Misr 2, Sids 1, Sids 11, Sids 12, Gemmeiza 7, Gemmeiza 9, Gemmeiza 11, Gemmeiza 12, Shandaweel 1, Sakha 68, Sakha 93, Sakha 94, Sakha 95, Benisuif 1, Benisuif 5, and Agaseed 22. Seed-borne mycoflora of the samples were detected in vitro using three seed-health testing techniques. A total of 44 fungal species belonging to 20 genera were identified from the wheat grains collected. Frequency (percentage of samples infected) and incidence (percentage of grains infected) of 44 fungal species per wheat growing governorate are presented in Table 1, and frequency and density (numbers of teliospores or embryos infected per 100 g grain), respectively, for two smut fungi are presented in Table 2. Among them, *Tilletia tritici* and *Alternaria alternata* were the most prevalent in all the wheat-growing governorates. *T. tritici* had the largest density, while *A. alternata* had the greatest incidence. Geographically, *T. tritici* teliospore densities recorded were greater in the southern governorates along the Nile valley, particulary Sohag, Bani Suef and Al-Menia, while the incidence of *A. alternata* was high in all wheat growing governorates in the Nile Delta and northern governorates except Alexandria. *Cladosporium* spp. and *Stemphylium* spp. followed *A. alternata* in terms of the incidence among wheat-pathogenic fungi identified. The southern governorates recorded a greater incidence of *Cladosporium* spp. than the other areas. Regarding two wheat smut fungi, *T. tritici* was identified in more governorates than *Ustilago tritici* (Table 2).

*Fusarium verticillioides* was the dominant species among the *Fusaria* isolated from wheat grains (frequency 84%), while *F. equiseti*, *F. fujikuroi*, *F. oxysporum*, and *F. thapsinum* were less frequently recorded. Regarding the genus *Bipolaris*, *B. sorokiniana* was the most prevalent species, while *B. austrostipae* and *B. hawaiiensis* were less prevalent. Among the 44 fungus species, many were known crop pathogenic fungi. Examples were *T. tritici*, *U. tritici*, *A. alternata*, *B. cynodontis*, *B. tetramera*, *F. proliferatum*, *F. equiseti*, *F. verticillioides*, *F. fujikuroi*, and *Exerohilum rostratum*. In addition, a range of post-harvest pathogenic fungi known to be with/without toxigenic backgrounds, such as *Penicillium* spp., *Nigrospora* spp., *Stemphylium globuliferum*, *Trichothecium roseum*, *Cladosporium anthropophilum*, *C. mebaldsii*, *Epicoccum purpurascens*, and *Rhizopus* spp. was also isolated.

### 3.2. Biodiversity of Wheat Seed-Borne Fungi

**Figure 2:**
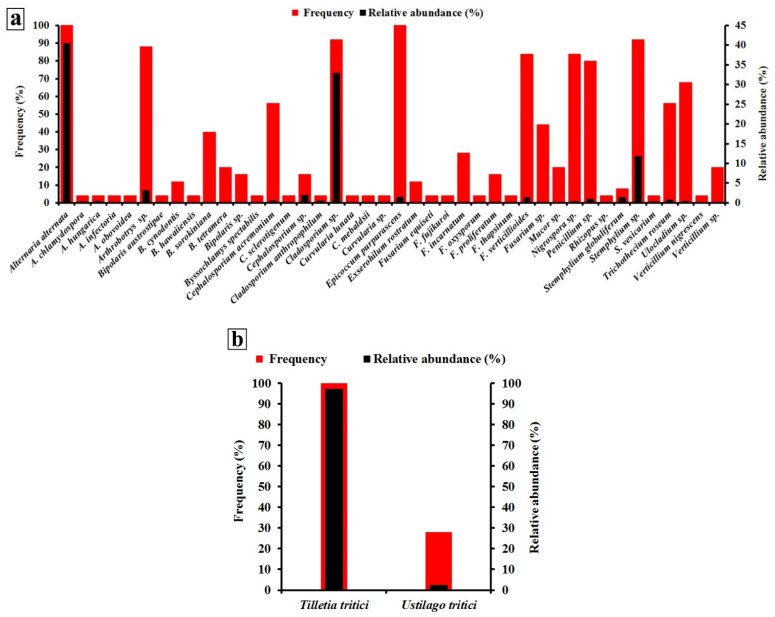
Frequency (%) and relative abundance (%) of wheat seed-borne fungus species detected with the deep-freezing blotter method (**a**) and two wheat smut fungi detected using the washing test for *T. tritici* and embryo count test for *U. tritici* (**b**) aggregated across all 25 wheat-growing governorates of Egypt.

Relative abundance and frequency of the aggregated seed-borne fungus species across all 25 wheat-growing governorates are illustrated in Figure 2a,b. Among the fungi identified in Figure 2a, *A. alternata* recorded the greatest relative abundance (40.4%), while *A. alternata* and *E. purpurascens* were the most frequent species (100% for each). Among the two smut fungi of wheat, *T. tritici* was more frequent (detected in 100% of samples collected) and most abundant (detected in 97.2% of seeds tested) (Figure 2b).

**Figure 3:**
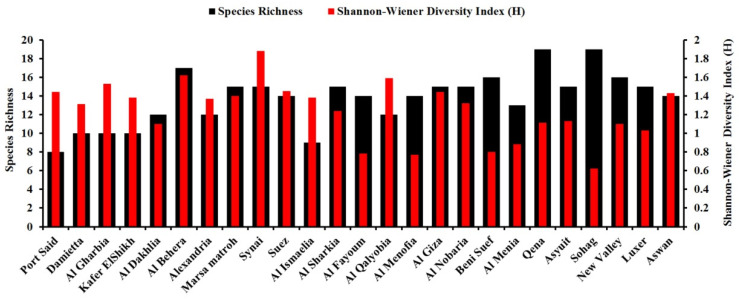
Species diversity (the Shannon–Wiener diversity index) and species richness (total number of fungus species identified) of the seed-borne fungus species identified in grain samples from each of the 25 wheat-growing governorates in Egypt.

**Figure 4:**
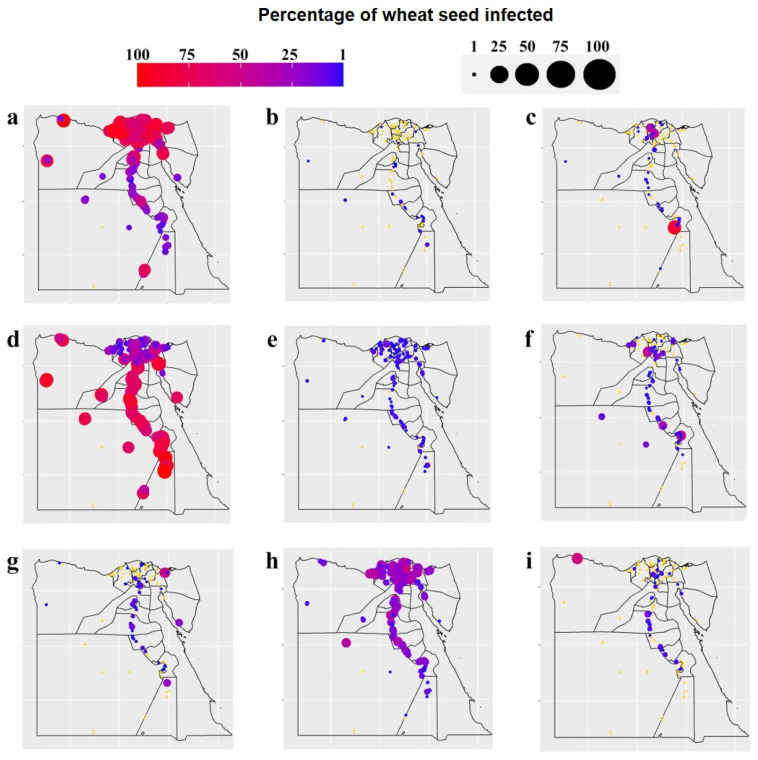
Geographical distribution and incidence (%) of important wheat seed-borne fungi in grain samples from each of the 25 wheat-growing governorates in Egypt: (**a**) *Alternaria alternata*, (**b**) *Bipolaris sorokiniana,* (**c**) *Cephalosporium acremonium,* (**d**) *Cladosporium* spp. (**e**) *Epicoccum purpurascens*, (**f**) *Fusarium verticillioides*, (**g**) *Penicillium* spp., (**h**) *Stemphylium* sp., and (**i**) *Trichothecium roseum* (circles in yellow mean no occurrence of the pathogen).

**Figure 5:**
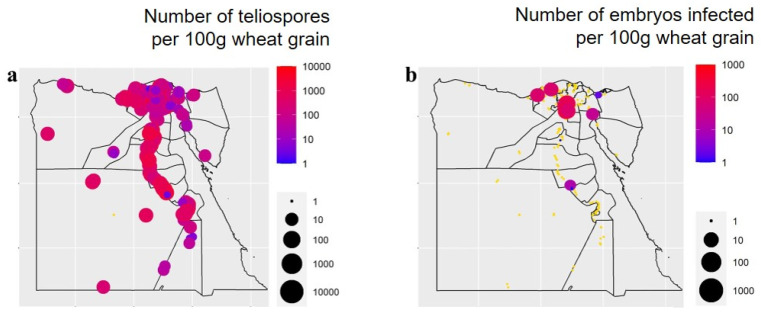
Geographical distribution and density (number of teliospores or infected embryos per 100 g grain) of wheat smut fungi (*Tilletia tritici* and *Ustilago tritici*, respectively) in grain samples from each of the 25 wheat-growing governorates in Egypt. (**a**) *Tilletia tritici*, and (**b**) *Ustilago tritici* (circles in yellow mean no occurrence of the pathogen).

Species diversity (i.e., the Shannon–Wiener diversity index) and richness of the seed-borne fungi in each of the 25 governorates surveyed are represented in Figure 3. A considerable variation in fungal species was observed among the governorates studied. Sinai governorate recorded the greatest species diversity (1.88), followed by Al-Behera, while the smallest species diversity (0.62) was recorded for Sohag. In addition, the greatest species richness was in Qena and Sohag governorates (19 species for each), while the smallest species richness was observed in Port Said (8 species). Distribution and incidence or density of the important pathogenic seed-borne fungi of wheat were mapped geographically (Figure 4 and Figure 5). The two main pathogens with a large incidence per governorate (with an incidence >40% of wheat seeds infected for 14 and 11 governorates out of 25, respectively) were *A. alternata*, with a greater incidence in the north of Egypt and Nile Delta, and *Cladosporium* spp., with a smaller incidence in the Nile Delta and Sinai areas. *Stemphylium* spp. was the third most common pathogen, but with a small incidence all over Egypt (with an average incidence *c.* 15% of wheat seeds infected), slightly larger in the Nile Delta. *Ustilago tritici* was present in only seven of the governorates, and mainly in Al-Qalyobia governorate.

### 3.3. Phylogenetic Analysis

**Figure 6:**
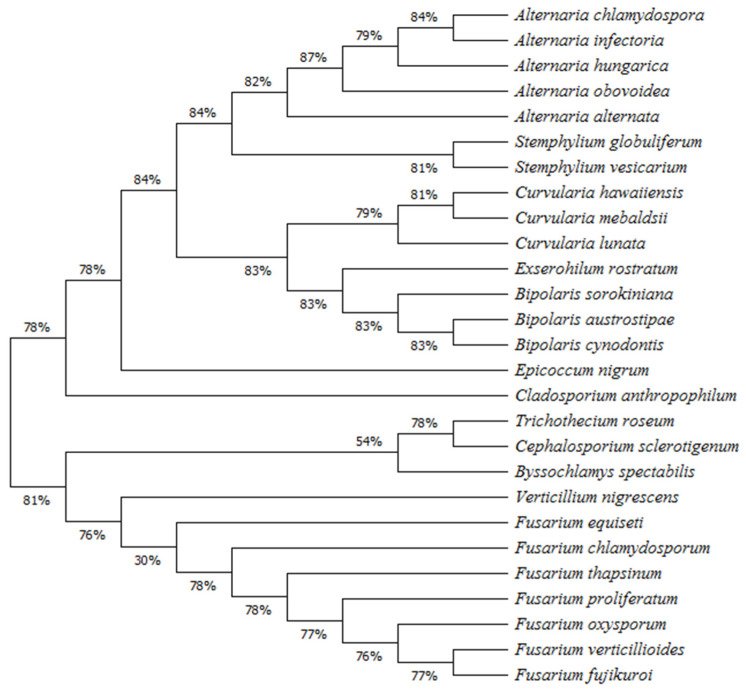
Phylogenetic tree of the seed-borne fungal species identified on wheat grain samples in all 25 wheat-growing governorates of Egypt.

Based on the molecular data of the seed-borne fungi isolated, a phylogentic tree was generated to identify their clustering structure and to understand their phylogenetic relationships (Figure 6). The results obtained demonstrated that all species isolated (27 spp.) came from one ancestor and are divided into two main groups based on genetic similarities. The first group showed 78% genetic similarity between its members. This group included 16 fungal species belonging to seven genera, while the other main group showed 81% similarity between its members and included 11 species belonging to five genera. The first main group contained two clusters; one of them contained one outgroup fungal species (*C. anthropophilum*), while the other branched into two sub-clusters: one of them included 14 species belonging to five closely related genera (*Alternaria*, *Stemphylium, Curvularia*, *Exserohilum*, and *Bipolaris*), while the other sub-cluster included one outgroup species (*E. purpurascens*). In the second main group, two clusters are present. One of them contained three species belonging to three closely related genera (*T. roseum*, *Cephalosporium sclerotigenum*, and *Byssochlamys spectabilis*), while the other cluster contained eight species belonging to two genera (seven species of the genus *Fusarium* and one species of *Verticillium*).

### 3.4. Pathogenicity Test

Fifteen pathogenic species from the seed-borne fungi isolated were subjected to pathogenicity tests to investigate their ability to infect and kill wheat seedlings (Table 3). Results showed that most of these 15 pathogenic fungal species had the ability to cause damping-off symptoms to varying degrees. In this regard, *B. cynodontis* caused the greatest percentages of pre- and post-emergence damping-off symptoms (25% and 12.5%, respectively), followed by *F. proliferatum* (22.5% and 15%, respectively) and *F. equiseti* (20% and 10%, respectively) when compared with the uninoculated control treatment. Two months after planting, the greatest seedling mortality was caused by *B. cynodontis* (37.5%), followed by *F. proliferatum* (35%) when compared with the uninoculated control seedlings. The smallest percentage mortality was recorded for *A. alternata* and *F. oxysporum* (2.5% each).

### 3.5. Correlations between the Occurrence of Wheat Fungal Pathogens and Weather Variables

**Figure 7:**
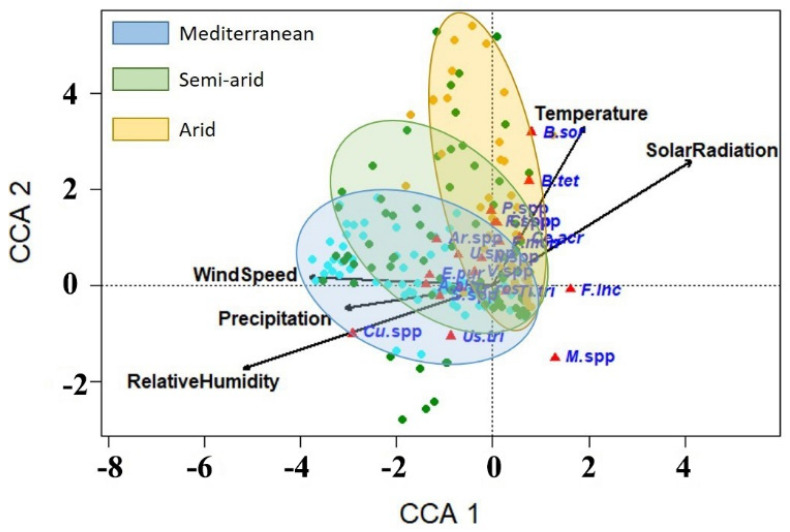
Ordination diagram of canonical correspondence analysis of seed-borne fungi in wheat grain samples from 25 wheat-growing governorates with five explanatory weather variables. Red triangles are fungal pathogens and arrows are weather variables. *A. alt* = *Alternaria alternata, Ar.* spp *= Arthrobotrys* spp*., B. sor* = *Bipolaris sorokiniana*, *B. tet* = *Bipolaris tetramera*, *Ce. acr* = *Cephalosporium acremonium, Cl.* spp *= Cladosporium* spp.*, Cu.* spp *= Curvularia* spp.*, E. pur* = *Epicoccum purpurascens, F. inc = Fusarium incarnatum, F.* spp = *Fusarium* spp.*, F. ver = Fusarium verticillioides, M*. spp = *Mucor* spp*., N.* spp *= Nigrospora* spp*., P.* spp *= Penicillium* spp*., S.* spp *= Stemphylium* spp.*, Ti. tri = Tilletia tritici, Tr. ros = Trichothecium roseum, U.* spp = *Ulocladium* spp., *Us.* tri = *Ustilago tritici*, and *V.* spp = *Verticillium* spp. Each small solid circle represents a sampling site where a pathogen was detected and grouped into climatic zones of Egypt: Mediterranean in blue, semi-arid in green, and arid in yellow.

Using the canonical correspondence analysis (CCA), correlations between the incidence of wheat seed-borne mycoflora and weather variables were determined (Figure 7). Results of ordination of CCA are presented in Table 4. The eigenvalues of the first four axes of CCA ordination were small, suggesting a well-structured data set and indicating stability of the ordination. The first three axes explain 92.7% of the species variance in the data set. The eigenvalues are a much better measure of the quality of the ordination and of the strength of the species–environment relationship than species–weather correlations, which were *r* = 0.57 for axis 1 and *r* = 0.42 for axis 2. The values for correlations between the weather variables in the growing season are presented in Table 5. The results showed that the mean temperature had a good positive correlation with the solar radiation (*r* = 0.61), and strong negative correlations with the relative humidity, precipitation, and wind speed (−0.57, −0.51, and −0.52, respectively). By contrast, the relative humidity exhibited a strong negative correlation with solar radiation (*r* = −0.87) and positive correlations with precipitation and wind speed. However, precipitation had a positive correlation with wind speed, but a negative correlation with solar radiation. In addition, there was a negative correlation between wind speed and solar radiation (*r* = −0.46). The CCA ordination diagram (Figure 7) shows the relative positions of the fungal species studied along the gradients of five weather variables, namely temperature, relative humidity, precipitation, wind speed, and solar radiation, in which the positions of fungal species are represented by red triangles, and the weather variables represented by long arrows. Length of the arrow refers to the effectiveness of the weather variable in the data interpretation, while direction of the arrow points to the maximum change in the weather variable. Results from CCA indicate that the relative humidity was the most influential weather variable, followed by temperature, solar radiation, wind speed, and precipitation, respectively. The CCA ordination diagram shows that *B. sorokiniana, B. tetramera, F. proliferatum, F. verticillioides*, and *C. acremonium*, located in the top right quadrant of the tri-plot, showed a positive correlation with temperature and solar radiation gradients. The seed-borne fungi *F. incarnatum, T. tritici*, and *Mucor* spp. are located in the lower right quadrant, showing little correlation with solar radiation or relative humidity, and very little correlation with other weather variables. However, *T. tritici* was more correlated with low values of solar radiation, temperature, and relative humidity than *F. incarnatum*, and *Mucor* spp. In the lower left quadrant, *Curvularia* spp., *Stemphylium* spp., *T. roseum*, and *U. tritici* showed good correlations with intermediate levels along the precipitation and relative humidity gradients, and to a lesser degree with the wind speed gradient. *A. alternata* and *E. purpurascens* showed good correlations with the wind speed gradient and to a lesser degree with the precipitation and relative humidity gradients. The fungal species *Penicillium* spp., *Cladosporium* spp., *Arthrobotrys* spp., *Ulocladium* spp., *Nigrospora* spp., and *Verticillium*. spp. showed positive correlations with wind speed and temperature, and little correlation with the other variables.

## 4. Discussion

From the plant pathological point of view, weather conditions have a great significance, being a corner of the disease triangle due to the vital effects that they have on pathogen growth, reproduction, dissemination, pathogenicity, life cycle, plant growth and susceptibility, and the plant–pathogen interactions. In the present study, we investigated the biodiversity and distribution of the seed-borne fungi of wheat, and their correlations with different weather variables in Egypt.

Phytopathogenic fungi can cause substantial yield losses in many economic crops. Some seed-borne fungal pathogens are very destructive, causing highly damaging diseases [27]. Moreover, some of them can produce mycotoxins as secondary metabolites, affecting human and livestock health [4]. Thus, data from the present study showed that 44 species belonging to 20 genera of seed-borne fungi were isolated and identified from wheat grains collected from 25 wheat-growing governorates in Egypt. Among them, *T. tritici*, the causal agent of stinking smut of wheat, and *A. alternata*, the causal agent of black point of wheat, were nationally the most prevalent across all wheat-growing governorates (see Figure 2a,b). Stinking smut of wheat is known to have a worldwide distribution, causing up to 50% yield losses in cases of severe disease and favorable conditions [28]. Association of wheat grain with *A. alternata* has been reported in many countries in the world suggesting its wide distribution. Their risk is, however, not due to yield loss they cause, but to reduction in the grain quality (discoloration) and nutritive value. Moreover, this fungus is known for production of health-hazardous mycotoxins such as alternariol, altenuene, and tenuazonic acid with mutagenic and genotoxic effects [29].

Results obtained revealed that wheat grains were associated with several pathogenic and toxigenic fungi, in either crops or post-harvest, such as *Fusarium* spp., the main source of mycotoxin contamination of wheat grains [30]. An array of mycotoxins reported to be produced by *Fusarium* spp. on wheat grain include fumonisins, deoxynivalenol, zearalenone, and nivalenol, in addition to the masked mycotoxins [31]. Furthermore, presence of high intensities of smut fungi (*T. tritici* and *U. tritici*) is of a great importance owing to their damaging effects on wheat yield, even if they occur at low intensity and frequency. Abrupt weather changes during the growing season can cause a shift in the geographical distribution of a pathogenic fungus, or it may alter its biology, leading to dramatic changes in the magnitude of the plant disease severity, resulting in more economic losses. For example, fluctuation in air temperature or humidity may make the conditions more suitable for aggressive pathogenicity or fungal growth [32]. Moreover, climate change may affect the ability of the fungus to produce mycotoxins. The geographical distribution of the pathogenic fungi reported in this study indicates a need for regular monitoring of the seed-borne pathogenic fungi, for example, every growing season if possible. In addition, developing new control measures against these pathogens is also needed.

Our results indicated that the sampled wheat grains represented twenty types of Egyptian wheat cultivars. In Egypt, development of wheat cultivars started in 1914 through the release of some domestic cultivars. In 1942, when stem rust threatened the Egyptian wheat, hybrid wheat programs began to breed new rust-resistant cultivars. The Egyptian hybrid programs have continued to release new cultivars with improved agronomic characteristics such as salinity tolerance, short plant height, high yield, water deficit resistance, early maturity, and more tillers. Of all wheat pathogenic fungi, the released cultivars by the Egyptian breeders up to date have been evaluated only for their resistance to rusts due to their severe threat to the wheat crop in Egypt [33]. For each wheat-growing governorate, specific wheat cultivars have been recommended by the Egyptian Ministry of Agriculture and Land Reclamation depending on the cultivar’s suitability to the climatic conditions in a given governorate in order to achieve the highest wheat productivity per unit area. Therefore, we cannot confirm that wheat cultivar type affected biodiversity of the detected seed-borne fungi.

Biodiversity of the fungal species in the individual wheat-cropping governorates was investigated using the Shannon–Wiener diversity index. This index indicates the distribution level of fungi among the species and their abundance in the governorates studied. Biodiversity data in the present study showed that Sinai governorate recorded the greatest species diversity, while the greatest species richness was in Qena and Sohag governorates. These governorates have semi-arid to arid climates with relatively high temperatures and moderate humidity and wind speed levels, which are suitable for the growth and dispersal of a wide range of fungi. This may explain the biodiversity and species abundance in these governorates.

Phylogenetic relationships among 27 fungal species belonging to 12 genera were investigated in this study. The main groups and their branched clusters showed high bootstrap values (>70%) indicating highly significant relations between members of each cluster/clade. The maximum likelihood analysis based on ITS sequences indicated that five *Alternaria* spp., namely *A. alternata, A. chlamydospora, A. hungarica, A. infectoria,* and *A. obovoidea*, are grouped in one clade, in which *A. alternata* (*Alternaria* section) is separately grouped in a sub-clade and the other species are grouped in another sub-clade. In addition, *A. infectoria* and *A. chlamydospora*, which belong to the closely related sections *Infectoriae* and *Phragmosporae*, respectively, are grouped together. This result is in agreement with that obtained by Somma et al. [34] who found that 164 strains of *Alternaria* spp., isolated from black point-infected-wheat grains, were grouped into two main clusters (the *A. alternata* species-section and the *A. infectoria* species-section) based on the phylogenetic analysis. Definite taxonomic classification of *Alternaria* spp. is not completely established yet. In some cases, molecular phylogeny may not be conclusive in clarifying the species boundaries due to the high degrees of similarity between them [35]. However, recent taxonomic investigations classified *Alternaria* spp. into 27 phylogenetic sections, and indicated that *A. infectoria* species-section (25 species) are very distant from that of *Alternaria* species-sections [36]. On the contrary to members of the *Alternaria* species-sections, species belonging to the *Infectoriae* section are characterized by production of the sexual stage, absence of the mycotoxin-producing ability, and production of specific secondary metabolites [37]. Our results revealed that both *S. globuliferum* and *S. vesicarium* are closely related to *Alternaria* spp., but phylogenetically they were distinct from each other. This result is consistent with the finding obtained by Habib et al. [38].

Our phylogenetic analysis showed that the first main group also included another separate and well-supported clade, which included three *Curvularia* spp. in one sub-clade and three *Bipolaris* spp. and *E. rostratum* in the other sub-clade. In addition, two outgroup species (*E. nigrum* and *C. anthropophilum*) were also included. This result is in agreement with that reported by Connally et al. [39] which separated the genera *Curvularia* and *Bipolaris* into two supported groups. Both genera were originally separated from *Helminthosporium* link based on the germination and morphology of their conidia. Morphological identification of their species is obscure, where a change in their culturing conditions may result in an inconsistent conidial morphology [40]. For example, morphological differentiation between *C. hawaiiensis* and *B. cynodontis* is difficult due to the overlapping in their conidial morphology, but the combined phylogenetic analyses using different genes could successively help in this concern [41]. Moreover, our results indicated that *E. rostratum* is closely related to *Bipolaris* spp., but phylogenetically distinct from them. The genera *Bipolaris* and *Exserohilum* can be morphologically differentiated from each other based on the morphology of the conidial hilum [42].

The second main group in our phylogenetic tree included two distinct clusters; one of them included seven species of the genus *Fusarium* and one species from genus *Verticillium,* while the other cluster included three species from different genera. Although the seven identified *Fusarium* species are included in a monophyletic sub-cluster, they represent different *Fusarium* sections. While, *F. oxysporum* belonged to the section *Elegans*, *F. equiseti* belonged to the section *Gibbosum*, and *F. chlamydosporum* belonged to the section *Sporothrichiella*, all other four species, namely *F. verticillioides*, *F. proliferatum, F. fujikuroi*, and *F. thapsinum*, belonged to the section *Liseola*. However, the recent taxonomic investigations indicated that section *Liseola* was paraphyletic [43]. This may explain the sub-grouping of the seven *Fusarium* spp. into different clades in the same sub-cluster. Our results are consistence with that obtained by Chala et al. [44] on *Fusarium* spp. associated with sorghum and finger millet grains. Species of the genus *Fusarium* are classified into 16 sections based on the presence/absence and shape of their micro-, macroconidia, and chlamydospores. Despite the advanced molecular phylogenetic analyses performed on *Fusarium* spp., many taxonomic relationships among them are still unclear [45].

In general, Egypt has an arid to semi-arid desert climate, which is characterized by high temperature with moderate relative humidity and low rainfall. The country is mainly in the subtropical area, but the southern part is tropical. Overall, there are four climatic regions in Egypt and three of them are represented by the locations where samples of wheat grains were collected in this study—Mediterranean in the northern governorates, semi-arid in the middle governorates, and arid in the southern governorates.

Using CCA, species–weather correlations of wheat seed-borne fungi were investigated in this study. Results from the CCA ordination diagram indicate that relative humidity was the most influential weather variable, followed by temperature, solar radiation, wind speed, and precipitation. This finding agrees with that of Mannaa and Kim [46] who reported that increased relative humidity and temperature increased growth of the rice seed-borne fungi (*A. candidus, A. flavus, A. fumigatus, P. fellutanum*, and *P. islandicum*). Humidity level is a critical limiting factor for biology of fungi, affecting their growth, reproduction, and pathogenicity in many ways. The growth of some fungi is favored by high humidity levels, while others prefer moderate humidity levels and some fungi even tend to develop better at relatively low humidity. For example, Li et al. [47] reported that mycelium of *Magnaporthe oryzae*, the cause of grey leaf spot of ryegrass, could not form appressoria when incubated at humidity levels ≤ 88%. Moreover, no conidiation was observed at humidity levels ≤ 92%, exhibiting the need of one fungus for two different humidity levels for infection, colonization, and sporulation. These findings show how the humidity can influence the biology and epidemiology of the fungus. Results from the CCA showed a good correlation of incidence of the pathogenic fungi *B. sorokiniana, B. tetramera, Fusarium* spp., including *F. incarnatum* (a synonym of *F. semitectum*) and *F. verticillioides* (a synonym of *F. moniliforme*), with air temperature. However, *B. sorokiniana* and *B. tetramera* were more closely correlated with high temperature than the others. These findings agree with those obtained by Cendoya et al. [48] who recorded the maximum growth rate of the fumonisin-producer fungus *F. proliferatum* on irradiated wheat grains at 25 °C, while the best temperature for fumonisin production was at 15–25 °C. These results indicate the critical effect of temperature on the growth rate and mycotoxin production of *F. proliferatum*. In a recent study, it was found that the maximum germination rate of *B. sorokiniana* was obtained at 30 °C, while the minimum was observed at 10 °C, showing the vital influence of relatively high temperature on their sporulation [49]. These findings agree with those obtained in the present study. Temperature can affect fungal growth by affecting the kinetics of the cellular enzymatic reactions and modulating the secretome of the cell [50]. On the other hand, a good correlation was observed for the smut fungus *T. tritici* with low levels of the solar radiation gradient. High solar radiation treatments have been used for the management of smut diseases of wheat [51], showing the negative effects of high levels of solar radiation on the fungal growth.

Our results revealed that incidences of *A. alternata* and *E. purpurascens* were closely correlated with high wind speeds, unlike incidence of *Verticillium* spp. that were correlated with low wind speeds. Wind is considered one of the major vectors of fungal spore dispersal, especially for *A. alternata*. Its conidia have morphological characteristics (shape and size), which are typical for wind-dispersed spores and support their long-distance wind dispersal and deposition leading to disease spread [52]. For instance, a recent study investigated the aerial spore concentration of fungal pathogens that escaped from infected fields and found that the rapid decrease in the air-borne spore concentration with height was not as frequently observed for *A. alternata* as for other pathogens such as *A. solani*. More interestingly, the method of production of *A. alternata* conidia (in chains) makes them easily detached by wind and facilitates their escape from the crop canopy [53]. Long-distance dispersal of fungal spores represents a vital survival approach for invading new regions and overwintering between agricultural seasons. In other words, their invasive potential depends on their ability to utilize the atmospheric conditions to spread and survive [52]. Results from CCA showed that *T. roseum* and *Stemphylium* spp. were positively correlated with the precipitation, while *Curvularia* spp. (including *Curvularia mebaldsii*) were found to be correlated with high relative humidity. Conidia of some fungi can be splashed by rain droplets or transported by wind onto healthy plants in the vicinity or in new areas. In addition, a high water content is necessary for conidial germination of some fungi, with full cell turgidity required to start germination [54].

The obtained results are of a great importance to improve our understanding and help to predict the future outbreaks of these pathogenic fungi in response to global climate change. Based upon these predictions, a set of adaptation and mitigation strategies should be considered by decision makers to minimize the expected disease spread including:

- Release of new resistant cultivars by wheat breeders;
- Reconsidering the date/location of wheat cultivation along the wheat-growing areas to avoid the disease spread;
- Use of heat tolerant biocontrol agents;
- Utilizing new disease control measures and tillage practices.

However, it should be noted that, although samples used in this study were carried out at many sites all over Egypt, the weather experienced by the wheat crop only covered one year. Over this short period, it is not possible to make a statement about weather-related trends during the wheat growing season for the whole country or a given region. In order to make general statements on trends in weather-related trends in seed-borne mycoflora of wheat, similar tests at national/governorate scales would have to cover a longer period of growing seasons. In the meantime, other factors, such as use and changes in cultivars and crop protection measures, should be considered.

## 5. Conclusions

Surveillance investigations for pathogenic and toxigenic fungi are important to refine our understanding of their epidemiology and help in predicting their outbreaks. This study provides baseline information on biodiversity, phylogenetic relationships, and geographical distribution of important pathogenic and toxigenic seed-borne fungi of wheat in April/May 2019 across wheat-cropping governorates of Egypt. In addition, it shows the correlations between occurrence of seed-borne fungal species and different weather variables. Fifteen out of the 44 fungal species identified are pathogenic and some of them are toxigenic. Their presence and prevalence need to be considered when implementing and developing crop disease protection strategies, such as increasing public awareness of seed health and testing seed quality for food security and food safety. Thus, pathogen surveillance, accurate diagnosis, and early warning programs should help to detect, prevent, and control wheat diseases in Egypt. The species that produce mycotoxin were identified. Relevant researchers should pay attention to these findings and alert food standard agents to warn food processors to look after consumers and animal feed producers and distributors. The findings about relationships between occurrence of seed-borne fungal species and different weather variables may be used in predicting disease occurrence and disease spread to uninfected areas. Moreover, our findings are useful for other wheat-growing countries that share the same climatic conditions such as Sudan, Algeria, Libya, Tunisia, Saudi Arabia, and Morocco.

This report is based on new research into the occurrence and distribution of seed-borne pathogens of wheat crops in a national scale survey in one season in Egypt. Together with the derived baseline frequencies and incidences of fungal species identified, it can be used to assess changes in occurrence and distribution of pathogens and detect new pathogens in the future, for example under climate change. These data provide improved understanding and preliminary results to study future outbreaks of these fungi in response to global climate change. Based upon these predictions, disease control measures, feasibility, suitability, and timing of wheat cultivation in particular regions may need to be reconsidered. Moreover, understanding of epidemics of wheat seed-borne fungi may be applied to other fungal pathogens, at least in the same genera, attacking other cereals, particularly in countries that share the same climate. However, more investigations and further data are needed to run climate change scenario simulations with these data. Weather-based disease models, disease-based yield loss models, and crop growth models for wheat should be developed and integrated. For developing these models, both historic and newly observed crop disease and crop yield data are necessary. In addition, education of wheat-growing farmers is necessary to prevent yield losses due to sudden outbreaks of crop diseases.

## Figures and Tables

**Table 1:** Frequency (F) and average incidence (I) of 42 wheat seed-borne fungus species in each of the 25 wheat-growing governorates of Egypt ^a^.

Fungus	Governorate ^a^																								-	-
	Port Said		Damietta		Al-Gharbia		Kafr El-Sheikh		Al-Dakahlia		Al-Behera		Alexandria		Matrouh		Sinai		Suez		Al-Ismaelia		Al-Sharkia		-	-
	F% ^b^	I% ^c^	F%	I%	F%	I%	F%	I%	F%	I%	F%	I%	F%	I%	F%	I%	F%	I%	F%	I%	F%	I%	F%	I%	-	-
*Alternaria alternata*	100	79.8	100	65.1	100	61.4	100	83.4	100	71.8	75	55.8	0	0	100	59.5	100	53.9	100	38.8	100	80.5	100	65.7	-	-
*A. chlamydospora*	0	0	0	0	0	0	0	0	0	0	13	8.2	0	0	0	0	0	0	0	0	0	0	0	0	-	
*A. hungarica*	0	0	0	0	0	0	0	0	0	0	13	10.4	0	0	0	0	0	0	0	0	0	0	0	0	-	-
*A. infectoria*	0	0	0	0	0	0	0	0	0	0	0	0	0	0	0	0	0	0	0	0	0	0	43	6.1	-	-
*A. obovoidea*	0	0	0	0	0	0	0	0	0	0	0	0	0	0	0	0	0	0	0	0	0	0	22	0.3	-	-
*Arthrobotrys* sp.	80	79.8	78	3.4	63	1.4	89	2.2	83	8.5	100	6.4	86	3.9	43	1.4	38	0.3	25	0.13	63	0.7	56	6.2	-	-
*Bipolaris cynodontis*	0	0	0	0	0	0	0	0	0	0	0	0	14	0.6	0	0	0	0	0	0	0	0	0	0	-	-
*B. sorokiniana*	0	0	0	0	0	0	0	0	0	0	13	0.1	0	0	14	0.1	0	0	0	0	13	0.1	0	0	-	-
*B. tetramera*	0	0	0	0	0	0	0	0	0	0	13	0.1	0	0	0	0	13	0.1	0	0	0	0	0	0	-	-
*Byssochlamys spectabilis*	0	0	0	0	0	0	0	0	0	0	0	0	0	0	0	0	0	0	0	0	0	0	22	0.2	-	-
*Cephalosporium acremonium*	0	0	22	0.2	25	4.8	22	0.2	0	0	25	0.12	0	0	14	0.1	0	0	0	0	0	0	14	1.1	-	-
*Cephalosporium* sp.	0	0	0	0	0	0	0	0	0	0	0	0	0	0	0	0	25	18.9	0	0	0	0	0	0	-	-
*Cladosporium anthropophilum*	0	0	0	0	0	0	0	0	0	0	0	0	0	0	0	0	0	0	0	0	0	0	71	13.9	-	-
*Cladosporium* spp.	100	18.2	100	15.4	100	21.9	100	10.2	100	9.6	100	6.6	86	10.6	100	62.3	88	19.4	100	80.5	100	30.7	0	0	-	-
*Curvularia* spp.	0	0	0	0	0	0	0	0	0	0	13	0.1	0	0	0	0	0	0	0	0	0	0	0	0	-	-
*Epicoccum purpurascens*	80	1.2	89	2.1	100	0.8	100	5.2	92	2.63	100	6.3	86	1.2	43	0.9	88	2.4	75	2	0	0	78	0.9	-	-
*Exserohilum rostratum*	0	0	0	0	0	0	0	0	0	0	0	0	0	0	0	0	0	0	25	0.13	0	0	0	0	-	-
*Fusarium equiseti*	0	0	0	0	0	0	0	0	0	0	0	0	0	0	14	0.3	0	0	0	0	0	0	0	0	-	-
*F. fujikuroi*	0	0	0	0	0	0	0	0	8	0.13	0	0	0	0	0	0	0	0	0	0	0	0	0	0	-	-
*F. proliferatum*	0	0	0	0	0	0	0	0	0	0	0	0	0	0	14	0.3	0	0	0	0	0	0	0	0	-	-
*F. verticillioides*	60	3.2	0	0	13	0.06	22	0.1	8	0.04	0	0	57	3.3	0	0	25	0.13	25	0.3	0	0	33	0.9	-	-
*Fusarium* spp.	40	0.6	33	0.2	0	0	11	0.06	0	0	13	0.2	0	0	0	0	13	0.1	0	0	13	0.1	0	0	-	-
*Mucor* spp.	0	0	0	0	0	0	0	0	0	0	0	0	0	0	14	0.1	0	0	0	0	0	0	0	0	-	-
*Nigrospora* spp.	0	0	11	0.3	13	0.06	0	0	8	0.04	25	0.2	29	0.3	29	0.4	0	0	50	0.3	38	0.3	22	0.17	-	-
*Penicillium* spp.	0	0	0	0	0	0	11	0.4	25	0.2	13	0.1	0	0	29	0.3	63	11.3	25	0.13	0	0	22	0.11	-	-
*Stemphylium globuliferum*	0	0	0	0	0	0	0	0	100	30.2	0	0	0	0	0	0	0	0	0	0	0	0	56	11.3	-	-
*Stemphylium* spp.	100	35.9	100	15.8	100	24.1	100	34.1	0	0	88	13.6	100	23	100	12.4	88	13	100	6.13	100	22.4	0	0	-	-
*S. vesicarium*	0	0	0	0	0	0	0	0	0	0	0	0	0	0	0	0	0	0	0	0	0	0	44	10.2	-	-
*Trichothecium roseum*	0	0	0	0	0	0	0	0	17	0.3	0	0	0	0	14	7.9	13	0.3	25	0.13	0	0	22	0.4	-	-
*Ulocladium* spp.	0	0	11	0.06	25	0.1	0	0	33	0.2	50	0.3	14	0.1	29	0.6	50	2.9	50	0.4	0	0	0	0	-	-
*Verticillium* spp.	0	0	0	0	0	0	0	0	0	0	0	0	14	0.3	0	0	13	0.3	0	0	0	0	0	0	-	-
**Fungus**	**Governorate**																									
	**Al-Fayoum**		**Al-Qalyobia**		**Al-Menofia**		**Al-Giza**		**Al-Nobaria**		**Bani Suef**		**Al-Menia**		**Qena**		**Assiut**		**Sohag**		**New Valley**		**Luxor**		**Aswan**	
	**F%**	**I%**	**F%**	**I%**	**F%**	**I%**	**F%**	**I%**	**F%**	**I%**	**F%**	**I%**	**F%**	**I%**	**F%**	**I%**	**F%**	**I%**	**F%**	**I%**	**F%**	**I%**	**F%**	**I%**	**F%**	**I%**
*Alternaria alternata*	100	52	100	70.7	100	67.9	100	22	100	80.1	100	22.6	100	15.8	100	17.3	100	30.8	100	14.9	100	30.7	100	7.8	100	25.7
*Arthrobotrys* spp.	38	0.7	75	4	75	1.8	43	0.3	88	26.1	0	0	0	0	13	0.1	25	1	38	1.4	25	1.9	0	0	25	11.8
*Bipolaris austrostipae*	0	0	0	0	0	0	0	0	0	0	0	0	0	0	25	0.2	0	0	0	0	0	0	0	0	0	0
*B. cynodontis*	0	0	0	0	0	0	0	0	13	0.13	0	0	0	0	25	0.3	0	0	0	0	0	0	0	0	0	0
*B. hawaiiensis*	0	0	0	0	0	0	0	0	0	0	25	0.13	0	0	0	0	0	0	0	0	0	0	0	0	0	0
*B. sorokiniana*	0	0	13	0.1	0	0	0	0	0	0	50	0.3	0	0	0	0	50	0.9	25	0.13	13	0.3	13	0.2	13	0.6
*B. tetramera*	0	0	0	0	0	0	0	0	0	0	13	0.1	0	0	38	0.5	0	0	0	0	0	0	13	0.1	0	0
*Bipolaris* spp.	0	0	0	0	0	0	0	0	0	0	0	0	25	0.2	0	0	0	0	13	0.13	0	0	13	0.1	25	0.13
*Cephalosporium acremonium*	13	0.1	0	0	25	0.4	43	1.3	13	0.7	0	0	63	0.9	50	0.7	13	0.1	50	0.8	0	0	0	0	0	0
*C. sclerotigenum*	0	0	0	0	0	0	0	0	0	0	0	0	0	0	0	0	0	0	0	0	25	0.3	0	0	0	0
*Cephalosporium* spp.	0	0	0	0	0	0	0	0	0	0	0	0	0	0	0	0	0	0	0	0	0	0	63	23.3	13	0.1
*Cladosporium* spp.	100	39.9	100	25.8	100	26.7	100	73.9	88	18.3	100	69.7	100	66.7	100	72.6	100	70.3	100	69.6	100	63.8	75	65.6	100	84.6
*Curvularia lunata*	0	0	0	0	0	0	14	0.14	0	0	0	0	0	0	0	0	0	0	0	0	0	0	0	0	0	0
*Cu. mebaldsii*	0	0	0	0	0	0	0	0	0	0	0	0	0	0	0	0	0	0	0	0	13	0.2	0	0	0	0
*Epicoccum purpurascens*	63	1.4	75	2.6	50	1.1	71	0.8	63	2.1	75	0.9	50	0.5	63	0.7	100	3	63	1.1	50	0.5	63	1.3	50	1.6
*Exserohilum rostratum*	0	0	0	0	0	0	14	0.2	0	0	0	0	0	0	0	0	0	0	0	0	0	0	0	0	0	0
*Fusarium incarnatum*	13	0.1	0	0	13	0.3	0	0	0	0	25	0.2	0	0	25	0.13	13	0.13	38	0.5	0	0	50	0.7	0	0
*F. oxysporum*	0	0	0	0	0	0	0	0	0	0	0	0	13	0.3	0	0	0	0	0	0	0	0	0	0	0	0
*F. proliferatum*	0	0	0	0	0	0	0	0	0	0	25	0.3	0	0	0	0	0	0	25	0.4	13	1.9	0	0	0	0
*F. thapsinum*	0	0	0	0	0	0	0	0	0	0	0	0	0	0	50	0.6	0	0	0	0	0	0	0	0	0	0
*F. verticillioides*	50	0.6	50	2.4	38	1.8	29	1.9	13	5.4	50	0.9	75	1.5	88	6.8	75	2.13	63	5.13	88	5.6	50	0.6	13	0.1
*Fusarium* spp.	38	0.3	0	0	25	0.25	14	0.2	13	0.3	0	0	0	0	0	0	13	0.1	0	0	13	0.3	0	0	0	0
*Mucor* spp.	0	0	0	0	0	0	0	0	13	0.3	25	0.2	0	0	0	0	0	0	13	0.1	0	0	13	0.1	0	0
*Nigrospora* spp.	50	0.5	25	0.13	38	0.4	29	0.4	13	0.13	25	0.13	63	1.1	50	0.3	38	0.7	13	0.1	25	0.5	0	0	25	0.13
*Penicillium* spp.	63	2.4	63	2.6	38	1.2	14	0.2	13	0.13	38	0.3	75	2.9	38	0.2	50	0.5	25	0.8	13	0.1	25	0.13	13	3.1
*Rhizopus* spp.	0	0	0	0	0	0	0	0	0	0	38	0.2	0	0	0	0	0	0	0	0	0	0	0	0	0	0
*Stemphylium* spp.	88	16.3	88	14.8	88	21.9	100	11.1	100	16.6	88	18.4	100	13.4	100	10.2	100	19.1	88	11.4	63	5.1	100	4.1	63	2.3
*Trichothecium roseum*	25	0.3	13	0.2	63	1.3	14	0.6	0	0	0	0	75	4.2	13	0.8	13	0.1	63	2.4	0	0	25	0.2	0	0
*Ulocladium* spp.	38	0.3	13	0.13	38	0.3	29	0.2	13	0.1	0	0	25	0.13	13	0.1	0	0	25	0.13	38	0.5	0	0	0	0
*Verticillium nigrescens*	0	0	0	0	0	0	0	0	0	0	0	0	0	0	0	0	0	0	0	0	0	0	13	0.2	0	0
*Verticillium* spp.	0	0	0	0	0	0	0	0	25	0.4	0	0	0	0	13	0.13	0	0	0	0	13	0.1	0	0	0	0

**^a^** Twenty-five wheat-growing governorates were surveyed during April–May in 2019 for wheat seed-borne fungi. For each governorate, four districts were selected; in each district, two villages in opposite directions were selected, and one wheat field from each village was sampled. Thus, eight wheat grain samples were collected from each governorate. For each sample, 400 grains were tested using a deep-freezing blotter method (DFB). ^b^ F = frequency of fungal species detected. F (%) = (number of seed samples in which a species occurred/total number of seed samples) × 100. ^c^ I = average incidence of a fungal species detected in a governorate (n = 8). I (%) = (number of seeds in which a species occurred/total number of seeds) × 100.

**Table 2:** Frequency (F%) and average density (D) of two wheat seed-borne smut fungus species in each of the 25 wheat-growing governorates of Egypt ^a^.

Fungus	Governorate ^a^																								-	-
	Port Said		Damietta		Al-Gharbia		Kafr El-Sheikh		Al-Dakahlia		Al-Behera		Alexandria		Matrouh		Sinai		Suez		Al-Ismaelia		Al-Sharkia		-	-
	F% ^b^	D ^c^	F%	D	F%	D	F%	D	F%	D	F%	D	F%	D	F%	D	F%	D	F%	D	F%	D	F%	D	-	-
*Tilletia tritici*	100	29	100	12	87.5	60	100	125	100	487	100	221	100	232	100	263	85.7	40	100	52	87.5	82	100	398	-	-
*Ustilago tritici*	0	0	0	0	0	0	0	0	0	0	12.5	10	14.3	11	0	0	12.5	1	50	8	0	0	0	0	-	-
**Fungus**	**Al-Fayoum**		**Al-Qalyobia**		**Al-Menofia**		**Al-Giza**		**Al-Nobaria**		**Bani Suef**		**Al-Menia**		**Qena**		**Assiut**		**Sohag**		**New Valley**		**Luxor**		**Aswan**	
	**F%**	**D**	**F%**	**D**	**F%**	**D**	**F%**	**D**	**F%**	**D**	**F%**	**D**	**F%**	**D**	**F%**	**D**	**F%**	**D**	**F%**	**D**	**F%**	**D**	**F%**	**D**	**F%**	**D**
*Tilletia tritici*	100	914	100	214	100	1000	100	89	100	399	100	837	100	660	87.5	419	100	464	100	1552	100	442	100	446	100	50
*Ustilago tritici*	0	0	87.5	19.6	0	0	0	0	0	0	12.5	1	0	0	0	0	0	0	37.5	2	0	0	0	0	0	0

^a^ Twenty-five wheat-growing governorates were surveyed during April–May in 2019 for wheat seed-borne fungi. For each governorate, four districts were selected; in each district, two villages in opposite directions were selected, and one wheat crop from each village was sampled. Thus, eight wheat grain samples were collected from each governorate. For each sample, 100 g of grains were tested using the washing method for detection of *Tilletia tritici* or embryo count method for *Ustilago tritici*. ^b^ F % = frequency of fungal detection. F (%) = (number of seed samples in which a species occurred/total number of seed samples) × 100. ^c^ D = average density of smut fungus in a governorate (n = 8) = (number of teliospores per 100 g of wheat grains) for *Tilletia tritici* and = (number of infected embryos per 100 g of grains) for *Ustilago tritici*.

**Table 3:** Pathogenicity of 15 seed-borne fungus species isolated *.

Fungus	Pre-Emergence Damping Off ** (%)	Post-Emergence Damping Off ** (%)	Survival ** (%)
Control (fungus-free)	1.0 ^e^	0.0 ^d^	99.0 ^a^
*Alternaria alternata*	2.5 ^e^	0.0 ^d^	97.5 ^ab^
*Bipolaris austrostipae*	7.5 ^c–e^	7.5 ^a–d^	75.0 ^c–f^
*B. cynodontis*	25.0 ^a^	12.5 ^ab^	62.5 ^f^
*B. sorokiniana*	12.5 ^b–e^	2.5 ^cd^	85.0 ^a–e^
*B. tetramera*	17.5 ^a–c^	5.0 ^b–d^	77.5 ^c–f^
*Cladosporium anthropophilum*	15.0 ^a–d^	2.5 ^cd^	82.5 ^b–e^
*Curvularia mebaldsii*	15.0 ^a–d^	0.0 ^d^	85.0 ^a–e^
*Exerohilum rostratum*	15.0 ^a–d^	0.0 ^d^	85.0 ^a–e^
*Fusarium chlamydosporum*	17.5 ^a–c^	10.0 ^a–c^	72.5 ^d–f^
*F. equiseti*	20.0 ^ab^	10.0 ^a–c^	70.0 ^ef^
*F. fujikuroi*	12.5 ^b–e^	5.0 ^b–d^	82.5 ^b–e^
*F. oxysporum*	2.5 ^e^	0.0 ^d^	97.5 ^ab^
*F. proliferatum*	22.5 ^ab^	15.0 ^a^	65.0 ^f^
*F. verticillioides*	5.0 ^de^	5.0 ^b–d^	90.0 ^a–c^
*Stemphylium globuliferum*	7.5 ^c–e^	5.0 ^b–d^	87.5 ^a–d^

* The seed-borne fungi isolated were tested for their pathogenicity using a soil infestation technique with fungus growing on sterilized sorghum–sand medium (1:1) at 10% moisture for 15 d at 26 ± 2 °C. Pots (25 cm-diameter) filled with sterilized soil were individually infested with the fungal inocula at 0.3%, mixed thoroughly, and watered with tap water and kept moistened for one week before planting. In each pot, 10 surface-sterilized wheat grains were sown. For each fungus, 10 pots (replicates) were used. All pots were arranged in a completely randomized way and kept for two months in a greenhouse. The pots were observed daily for grain germination, and the disease incidence was recorded. Each value is the mean of 10 replicates (pots), values within a column followed by the same letter(s) are not significantly different according to Duncan’s multiple range test (*p* ≤ 0.05). ** Pre-emergence damping off = seed/seedling death before emergence; Post-emergence damping off = seedling death after emergence; survival = living plants two months after planting.

**Table 4:** Results of ordination of the canonical correspondence analysis accounted for the first four axes.

Axis	1	2	3	4
Eigenvalue	0.104	0.037	0.015	0.009
Species–weather correlation	0.569	0.422	0.227	0.218
Cumulative percentage variance of species–weather relation	62.0	83.8	92.7	98.2

**Table 5:** Pearson moment correlations (*r*) matrix between five weather variables recorded from November 2018 to May 2019 inclusive in 25 wheat growing governorates of Egypt from which grain samples were collected (one weather station per governorate).

	Temperature	Relative Humidity	Precipitation	Wind Speed	Solar Radiation
Temperature	1 ^a^				
Relative humidity	−0.574 ***	1			
Precipitation	−0.511 ***	0.533 ***	1		
Wind speed	−0.520 ***	0.550 ***	0.275 **	1	
Solar radiation	0.609 ***	−0.874 ***	−0.336 ***	−0.456 ***	1

^a^ Values followed by ** or *** are significant at *p* ≤ 0.01 or *p* ≤ 0.001, respectively.

## Data Availability

The data that support the findings of this study are available on request from the corresponding author. The data are not publicly available due to privacy or ethical restrictions. However, the main data representing averages per governorate are available in the manuscript (Table 1 and Table 2).
